# Scaffolded role-play for cross-text integrative writing in elementary Chinese: a teacher-vetted AI-drafted materials classroom study

**DOI:** 10.3389/fpsyg.2026.1837762

**Published:** 2026-08-07

**Authors:** Min Hui

**Affiliations:** Yuyingli Primary School, Tianjin, China

**Keywords:** AI-drafted materials, cross-text integrative writing, elementary Chinese instruction, multi-text reading, scaffolded role-play

## Abstract

**Introduction:**

The *Chinese Language Curriculum Standards for Compulsory Education (2022 Edition)* emphasize multi-text learning through learning task clusters, yet classroom practice often continues to treat linked texts as separate reading lessons. This study examined an integrated instructional package for linked-text reading and cross-text writing in Grade 3 Chinese.

**Methods:**

A posttest-only classroom quasi-experimental design was adopted. Participants were two intact Grade 3 classes from a public primary school in Tianjin, China, with a valid analytic sample of 54 students. Both classes studied the same four thematic units with identical texts, instructional time, teacher, learning objectives, and posttest writing task. The intervention class received an instructional package combining teacher-vetted AI-drafted materials, scaffolded role-play, probing-question chains, evidence checkpoints, structured turn-taking, and cross-text writing. The comparison class received conventional reading instruction. Outcomes included a cross-text integrative writing task and a post-intervention learning-experience questionnaire.

**Results:**

The intervention class achieved a higher total writing score than the comparison class, Welch’s *t*(51.8) = 2.98, *p* = 0.004, *d* = 0.81. Supplementary ANCOVA controlling for midterm Chinese achievement showed a similar between-class difference, *F*(1, 51) = 8.72, *p* = 0.005, partial η^2^ = 0.146. Differences were concentrated in thematic integration, textual evidence, and comparison and transfer, whereas expressive structure showed no reliable difference. Students in the intervention class also reported higher engagement, evidence awareness, perceived integration and transfer, strategic confidence, and acceptability, together with lower perceived task difficulty and pressure.

**Discussion:**

The findings provide preliminary classroom evidence that an integrated instructional package may support evidence-aware cross-text writing and improve students’ learning experiences. Further multi-class studies incorporating pretests, delayed assessments, and classroom-process data are needed to examine the robustness and persistence of these effects.

## 1 Introduction

The *Chinese Language Curriculum Standards for Compulsory Education (2022 Edition)* organize Chinese language curriculum content through learning task clusters and require students to complete reading, communication, expression, and inquiry in authentic language-use contexts. For elementary Chinese reading instruction, the shift brought by the task-cluster orientation is not simply an increase in the number of texts to be read. More importantly, students are expected to work with relationships among multiple texts around a shared theme. When Grade 3 students read several texts under the same theme, they are usually able to state the main idea of a single text. However, they still need explicit classroom support to compare similarities and differences across texts and to turn dispersed details into evidence-based synthetic judgments. Rong et al. (2022) argue that the core function of learning task clusters lies in connecting reading, discussion, and expression through shared learning goals. Cross-text integration therefore becomes a key issue in elementary Chinese classrooms: how students move from understanding multiple texts to producing evidence-based written expression (Rong et al., 2022).

This study uses the term cross-text integrative writing to refer to students’ written expression after they have developed a synthesized understanding based on two or more related texts. This concept is related to research on multiple-document comprehension, cross-document integration, source-based writing, and synthesis writing. Research on multi-text comprehension emphasizes that, when constructing an integrated response, readers need to understand the content of each text, establish connections among texts, track source information, and coordinate content, evidence, and judgment in their expression (Perfetti et al., 1999; Rouet and Britt, 2011; Britt and Rouet, 2012; Stadtler and Bromme, 2014). Recent studies on cross-text literacy and source-based reading and writing further emphasize that integration involves comparison, source awareness, claim-evidence coordination, and synthetic expression (Du and List, 2020; Anmarkrud et al., 2022; McCarthy et al., 2022; Incognito and Tarchi, 2024). In relation to writing tasks in elementary Chinese classrooms, this study examines students’ cross-text integrative writing performance from four dimensions: thematic integration, textual evidence, comparison and transfer, and expressive structure. This study situates cross-text integration in a writing task following Grade 3 students’ reading of multiple thematically linked texts, focusing on how students select textual evidence, organize comparative relationships, and produce written responses with thematic coherence.

In lower-grade classrooms, the main difficulty of cross-text integration often lies not in whether students have read multiple texts, but in whether they can turn textual details into evidence that supports a claim. In actual teaching, multi-text reading is sometimes treated as the sequential explanation of several individual texts. Students may retell plots and express feelings during discussion, but they less often explain the relationships among texts. Recent studies on multiple-text comprehension and integration also show that reading goals, task cues, and the way students express cross-textual links in writing can shape how they select, compare, and organize information from different texts (Lee E. et al., 2024; Savvidou et al., 2025; Wei et al., 2025). In writing, students may list texts one by one, offer vague conclusions, or provide insufficient evidence. For Grade 3 students, cross-text integration requires a clear procedural pathway: returning to the text to confirm details, comparing relationships among texts, and then organizing discussion outcomes into written judgments. Writing was selected as the primary outcome because written responses can more clearly reveal students’ evidence selection, claim organization, and thematic synthesis. Existing research also supports the relationship between writing about reading content and improvement in reading comprehension (Graham and Hebert, 2011).

To help students complete this process, the present study used role-play to organize cross-text discussion. Role identities allowed students to enter specific textual positions, speaking turns provided an order for discussion, and probing tasks required students to return to the original texts to supply reasons. Research on drama-based instruction suggests that the effects of such activities depend on task design, alignment with instructional goals, teacher guidance, and implementation quality (Lee et al., 2014). In the classroom activities of this study, role boundaries, speaking turns, evidence checkpoints, and time allocation jointly constrained the scope of discussion. Students first stated a position based on their assigned roles, then searched for textual evidence in response to probing questions, compared relevant information across different texts, and finally turned the discussion outcomes into integrated written expression. This activity arrangement moved cross-text integration beyond general requirements such as “comparison” and “synthesis” and translated it into classroom steps that students could carry out. In the actual implementation, role cards were not used simply to assign performative identities. Instead, they specified which text, character position, or event perspective students should speak from. The probing-question chains required students to return to the original texts to locate support after presenting an initial view. The evidence checkpoints further prompted students to identify where their judgments were grounded in the textual content, while the turn-taking rules helped maintain an orderly discussion within limited lesson time. Through these arrangements, role-play was transformed into a text-evidence-oriented form of classroom discussion and was linked to subsequent cross-text comparison and written expression.

ChatGPT was used in this study only during the teacher-preparation stage. It mainly generated initial drafts of role cards, situational tasks, dialogue prompts, and probing-question chains. Students did not directly use the model during classroom learning. Considering that large language models may generate inaccurate, unverifiable, or developmentally inappropriate content, especially in educational contexts involving minors, AI-generated materials need to be reviewed and revised by teachers before entering the classroom (UNESCO et al., 2023). The teacher checked the AI-drafted materials in terms of textual accuracy, age appropriateness, value orientation, evidence prompts, and classroom pacing, and then incorporated the usable content into scaffolded role-play activities. In this study, AI served as a preparation support tool, while the selection, adjustment, and implementation of classroom activities were completed by the teacher. In actual use, the content generated by ChatGPT did not directly enter students’ learning process. Instead, it first served as candidate material for the teacher’s lesson preparation. Based on the textual content, Grade 3 students’ level of understanding, classroom time arrangements, and the need for evidence prompts, the teacher selected, deleted, revised, and reorganized these materials. They were then transformed into role cards, situational tasks, and probing questions within the role-play activities, supporting students in returning to the texts, explaining the basis of their judgments, and completing cross-text writing.

The study selected relevant texts from the 2019 nationally unified elementary Chinese textbook series and reorganized them into four thematic units, each comprising three thematically related reading texts. Two intact Grade 3 classes were taught by the same teacher, used the same learning materials, and followed the same instructional schedule, learning objectives, lesson duration, and posttest writing task. The intervention class adopted an integrated instructional package: the teacher first reviewed and revised the AI-assisted instructional materials, then organized scaffolded role-play supported by evidence checkpoints, probing-question chains, and structured turn-taking to guide students in evidence-based cross-text discussion and writing. The comparison class received conventional reading instruction. The study focused on organizing teacher-vetted AI-assisted material preparation, evidence-based role-play, structured text-source prompts, and cross-text writing into a continuous classroom teaching process, and examined the implementation of this approach in a regular elementary Chinese classroom. Using a posttest-only comparison of two intact classes, the analysis focused on context-specific between-class differences, including students’ cross-text integrative writing performance and their self-reported experiences of classroom engagement, evidence awareness, integration and transfer, strategy use, instructional acceptability, and perceived task difficulty and pressure.

## 2 Theoretical foundations

### 2.1 Cross-text integration and source-based writing

In elementary Chinese classrooms, cross-text reading is primarily intended to guide students to compare information across several texts within the same thematic unit and to explain how this information jointly supports a judgment. Recent studies on multiple-document learning have approached this issue from different angles: summarizing multiple documents, generating critiques, and defining criteria for evaluating integrated responses (McNamara et al., 2024; Primor and Barzilai, 2024). These studies point to the same instructional demand: students need to understand the main content of each individual text, identify similarities, differences, and relationships among texts, and incorporate relevant details into their own expression. In this study, cross-text integrative writing refers to students’ written expression based on multiple assigned texts. The task requires students to understand textual content, compare textual relationships, and develop a synthesized claim using evidence from the reading materials.

Developing an integrated understanding across several texts requires students to process both content relationships and source cues. Research on multi-text comprehension shows that, when readers process multiple texts, they need to connect information from different sources, attend to where information comes from, and coordinate textual content with source cues in their responses (Espinas and Wanzek, 2024; Lee Y. H. et al., 2024). Difficulties in multi-text learning often appear in two forms: students summarize each text separately and fail to form an overall understanding, or they make a general judgment without specifying the textual details on which the judgment is based (Kiili et al., 2020; Salmerón et al., 2020; Kullberg et al., 2022). In this study, cross-text integration mainly refers to students’ ability to identify a shared theme across several texts, compare relevant information, and support a synthesized judgment with textual evidence (McCarthy et al., 2022).

When cross-text integration becomes a writing task, students need to reorganize information gained through reading into claims and evidence. Source-based writing requires students to select information from texts, organize materials around a claim, and explain the relationship between evidence and judgment. For lower-grade students, evidence use does not simply mean locating a detail. It also involves judging whether the detail is relevant, explaining what claim the detail supports, and connecting that detail with information from another text. Research on source awareness and multiple-document literacy indicates that stronger source awareness and cross-text integration are associated with more coherent claim-evidence coordination in written responses (Anmarkrud et al., 2022; Incognito and Tarchi, 2024). Task design also shapes how students organize information. When a task explicitly requires comparison, synthesis, and evidence-based conclusions, learners are more likely to reorganize information from different texts and less likely to directly retell the content of a single text (List et al., 2024; Savvidou et al., 2025).

This study evaluates students’ cross-text integrative writing from four aspects: thematic integration, textual evidence, comparison and transfer, and expressive structure. Thematic integration examines whether students can form a shared thematic judgment across multiple texts. Textual evidence focuses on whether they can use specific details from the reading materials to support their claims. Comparison and transfer concerns how they explain similarities, differences, and task-relevant relationships among texts. Expressive structure assesses the clarity and coherence of the written response and the way the synthesized judgment is organized (Graham et al., 2020). These dimensions correspond to visible features in students’ written products and also reflect the extent to which they demonstrate source awareness, claim-evidence coordination, and synthetic expression.

### 2.2 Scaffolded role-play as text-anchored classroom discourse

Cross-text integrative writing requires students to propose claims, locate evidence, compare texts, and organize discussion outcomes into written judgments. In this study, role-play was used to organize this discussion process. Students entered specific textual positions through assigned roles, stated claims and responded to peers within speaking turns, and returned to the original texts to find support in response to probing questions. Drama-based instruction and role-based pedagogy have been associated with literacy outcomes, but their effects are influenced by task design, alignment with instructional goals, teacher guidance, duration, and implementation quality (Lee et al., 2020; Luo et al., 2024). Whether role-play can support cross-text writing depends on whether students’ speech remains centered on textual content, textual evidence, and relationships among texts.

When Grade 3 students engage in cross-text discussion, they need clear participation positions, question cues, and evidence-checking requirements. This design choice is consistent with recent classroom-discourse research that treats talk not as free discussion, but as an organized setting in which students revise ideas, build on peers’ contributions, and respond to teacher prompts that require reasoning or evidence (Bouwer et al., 2024; Nagpal et al., 2025; Tao et al., 2025). In the present study, scaffolded role-play was therefore designed as an activity process consisting of role cards, probing-question chains, evidence checkpoints, and speaking-time arrangements. Role cards specified the scope of students’ participation and connected each role with a textual perspective or task responsibility. Probing-question chains guided students from initial claims toward textual support and cross-text comparison. Evidence checkpoints required students to return to the assigned texts before confirming or revising a statement. Limits on speaking turns and time allocation ensured that discussion could be completed within regular lesson time. These arrangements were intended to reduce unsupported opinions, general retelling, and text-detached discussion, allowing students to move through the steps of making a claim, locating evidence, comparing texts, and forming a conclusion. Within this activity structure, role-play functioned as a way of organizing classroom discussion: through role allocation, probing prompts, and evidence checkpoints, it directed students’ talk toward textual evidence, cross-text comparison, and conclusion formation.

Scaffolding theory helps explain the role of role cards, probing questions, and evidence checkpoints in the classroom. Scaffolding refers to support that helps learners complete tasks they cannot yet complete independently and gradually makes the task structure available for learners’ own use. Recent reviews distinguish between pre-designed “hard scaffolds,” such as tools, prompts, and materials, and adaptive “soft scaffolds,” such as teacher questioning and contextualized classroom guidance (Dominguez and Svihla, 2023; Ertugruloglu et al., 2023). In this study, role cards, probing questions, and evidence prompts constituted pre-designed scaffolds. Role cards specified the textual positions from which students were expected to speak. Probing questions prompted students to return to specific plots, character actions, or textual statements as support. Evidence checkpoints required students to explain the correspondence between their claims and the textual content, while speaking-time arrangements maintained the sequence of discussion and the pace of the lesson. Teacher circulation, monitoring, and follow-up questioning provided immediate support by correcting text-detached statements and guiding students to add evidence and compare relevant information across texts. Research on document-based inquiry shows that external criteria, mapping tools, and guiding prompts can support source evaluation and evidence-based judgment, both of which are closely related to cross-text writing (Barzilai et al., 2025). For elementary students, cross-text synthesis involves the simultaneous demands of remembering textual content, judging the relevance of information, comparing texts, and organizing written expression. Scaffolded role-play translates these demands into executable classroom procedures through role allocation, responses to probing questions, evidence confirmation, and discussion organization.

### 2.3 Teacher-vetted AI-drafted materials as preparation support

Cross-text role-play activities require highly specific preparation materials. Teachers need to prepare role cards, situational descriptions, evidence prompts, sentence stems, and question chains, while also ensuring that these materials match the assigned texts, students’ age level, classroom time, and discussion sequence. Work on GenAI-supported instructional design has begun to treat these tools as aids for drafting lesson activities, inquiry tasks, and revision-oriented lesson-plan materials, with teacher judgment remaining central to selection and classroom adaptation (Moundridou et al., 2024). School-level research on GenAI integration also points to the need for clear guidelines, teacher competence, and context-sensitive implementation strategies when AI-generated materials enter educational practice (Cai et al., 2025). During lesson preparation, the teacher used ChatGPT to produce initial texts for role configurations, situational tasks, dialogue progression, and probing questions, which were then screened, deleted, revised, and reorganized. In processing these materials, the teacher focused on the assigned textbook content, Grade 3 students’ language-comprehension level, and the pace of classroom discussion. The usable parts were ultimately transformed into instructional materials for role cards, evidence prompts, and probing-question activities.

Elementary classrooms place high demands on the accuracy, appropriateness, and feasibility of AI-drafted materials. Foundation models may generate fabricated details, inaccurate interpretations, unclear source references, or language that does not match students’ developmental level. Elementary students usually lack the ability to identify errors or challenge unsupported statements; therefore, textual accuracy, age appropriateness, and classroom feasibility need to be checked by the teacher. Current discussions of risk management and governance also emphasize that, when generative AI is used in educational contexts, teacher supervision, source checking, age-appropriate adaptation, and privacy protection are necessary (Sahoo et al., 2024). During material review, the teacher adjusted the AI-drafted materials mainly in terms of alignment with the texts, students’ level of understanding, value-related expression, evidence prompts, and classroom pacing. Role settings needed to correspond to characters, events, or thematic relationships in the assigned texts. The language needed to match Grade 3 students’ comprehension and expression levels. Probing questions needed to guide students back to specific textual content, and the activity steps needed to fit the time constraints of regular lessons. Based on these judgments, the teacher removed content that had weak connections with textual evidence or contained excessive information, compressed dialogue turns, revised expressions that might affect students’ value understanding, and added evidence checkpoints at points of role speech and discussion transition. Prompts that originally encouraged open discussion were rewritten as questions requiring students to first locate textual details and then make judgments based on them.

Only after teacher review were the AI-drafted materials incorporated into the classroom design of scaffolded role-play. Role cards, situational tasks, probing questions, and evidence prompts were used to organize students’ cross-text discussion. AI provided initial material drafts, while the teacher adjusted them according to textual content and students’ characteristics. Classroom activities were organized around role-based speech, textual verification, comparative discussion, and written expression. The role of generative AI was mainly located in the preparation stage of instructional materials; the classroom implementation of scaffolded role-play still depended on the teacher’s judgment of texts, students, and instructional pacing.

## 3 Materials and methods

### 3.1 Participants and design

This study adopted a posttest-only, class-level quasi-experimental design. The participants were two intact Grade 3 parallel classes from a public primary school in Tianjin, China, and students were not individually randomized. The study was conducted within the school’s regular Chinese language curriculum and retained the existing class structure and teaching arrangements. The two classes were taught by the same Chinese language teacher, studied the same texts, and followed the same instructional schedule, learning objectives, lesson duration, and posttest writing task. The intervention class received scaffolded role-play instruction supported by teacher-reviewed AI-drafted materials, whereas the comparison class received conventional reading instruction.

The initial sample included 56 students, with 28 students in the intervention class and 28 students in the comparison class. The students were aged 8–9 years, including 29 boys and 27 girls. Students were included in the main analyses if they attended the core instructional sessions and completed both the posttest writing task and the learning-experience questionnaire. Students who missed core instructional sessions or had incomplete data were excluded from the corresponding analyses. The final valid analytic sample consisted of 54 students, with 27 students in the intervention class and 27 students in the comparison class.

Because the two intact classes may have differed in their prior learning background, classroom climate, and reading experience, students’ midterm Chinese language scores were collected before the instructional sequence. These scores were used to describe the two classes’ general Chinese language achievement and were included as a student-level covariate in supplementary analyses. The midterm score reflected students’ overall performance in Chinese language learning and provided contextual information for interpreting the posttest results. The school’s routine assessment system did not include a prior measure corresponding to the cross-text integrative writing task used in this study, so prior writing performance was not used as a direct baseline indicator. Students’ cross-text integrative writing performance was assessed using the unified posttest writing task administered after the full instructional sequence.

### 3.2 Ethics, consent, and data protection

Data collection was conducted within the school’s regular Chinese language teaching and classroom assessment activities. Before implementation, the school approved the study at the classroom-management level because the writing task and learning-experience questionnaire were embedded in routine classroom instruction, learning reflection, and post-instruction assessment. Parents or legal guardians were informed through the school’s established home–school communication channels about the purpose and procedures of the study, the types of data collected, the voluntary nature of participation, the anonymization procedures, and the intended use of the data for research reporting. Written informed consent for participation in the study was obtained from the participants’ legal guardians before data collection. The guardians were informed that participation was voluntary and that they could withdraw their child’s data from the research analyses without any academic or other disadvantage. Students were also provided with an age-appropriate explanation of the study procedures and were informed that they could decline to answer any questionnaire item or discontinue participation without penalty.

Before the questionnaire was administered, the teacher explained its purpose using language appropriate for Grade 3 students and clearly informed them that the questionnaire was intended to understand their learning experiences after the instructional sequence. Students were told that their responses would not affect their Chinese language grades, classroom evaluation, or relationship with the teacher. They could leave any item unanswered without academic disadvantage. Before data anonymization and analysis, no parent or legal guardian requested that their child’s data be excluded. The writing task was completed in class after the full instructional sequence. Students completed the learning-experience questionnaire using their parents’ mobile devices. When assistance was needed, parents or legal guardians were asked to provide only technical or reading support, such as opening the questionnaire link, reading item wording aloud, or explaining individual words in a neutral manner. They were not asked to suggest answers, evaluate students’ responses, or select options on behalf of the students. The questionnaire findings were therefore analyzed and interpreted as students’ post-intervention self-reported classroom experiences.

Before data processing, students’ names, class identifiers, and other potentially identifying information were replaced with anonymized codes. The final analytic dataset contained no student names, family information, contact details, or other personally identifiable information. The textbook materials used in classroom teaching were from the officially adopted nationally unified elementary Chinese language textbook series and were provided through the school’s regular textbook-use channels. The textbooks were used as part of ordinary classroom instruction, and the teacher consulted the printed textbook materials locally during lesson preparation. During AI-assisted lesson preparation, only instructional design information was provided to ChatGPT, including textbook titles, instructional objectives, teacher-generated content cues, role requirements, script-format requirements, and evidence checkpoints. Full textbook passages, scanned textbook pages, long verbatim excerpts, or complete reproductions that could substitute for the copyrighted textbook content were not entered into ChatGPT prompts. Student lists, individual learning records, parent information, questionnaire responses, writing scores, and identifiable samples of student writing were also not entered into ChatGPT prompts. Because ChatGPT is a third-party service and prompt content may be processed or stored by the service provider, the study avoided entering student data and full copyrighted textbook content into the system. AI-generated drafts were reviewed, revised, and stored locally by the teacher before classroom use. The anonymized writing scores and questionnaire data were used solely for the analyses and reporting presented in this study.

### 3.3 Instructional materials and classroom procedure

The instructional materials were selected from the 2019 nationally unified elementary Chinese language textbook series. The relevant texts were reorganized into four vertical thematic units, with each unit consisting of three linked texts. All four units were developed around the broad theme of “experience and wisdom,” but the texts differed in content focus, genre, narrative perspective, and mode of explanation. During reading, students were expected to compare similarities and differences in characters’ experiences, event development, expressive focus, or explanatory content, and to use these comparisons in the subsequent expression task. Each thematic unit contained a thematic line and an evidence line. The thematic line guided students to understand the three texts under a shared question, whereas the evidence line directed students’ attention to textual details that could support thematic judgments, including scene descriptions, character actions, psychological changes, narrative turning points, or explanatory information. In the classroom expression stage, students were required to identify the text from which each piece of evidence came and explain how that evidence supported their judgment. Before implementation, the teacher reviewed the four thematic units in terms of text length, reading difficulty, thematic complexity, and opportunities for cross-text comparison. The text combinations and integrative objectives of the four thematic units are shown in Table 1.

**TABLE 1:** Three linked texts and integrative objectives for four vertical thematic clusters.

Thematic cluster	Theme title	Three linked texts	Integrative objective
1	Wisdom and action on the waterways	*A Night Mooring by Maple Bridge; Borrowing Arrows with Straw Boats; Venice’s Little Boat*	Integrate strategies and judgment across different contexts under the shared theme of “action and wisdom.”
2	Observation: the starting point of knowing the world	*Ode to the Twilight River; Wang Rong Refuses the Plums by the Roadside; The Squirrel*	Extract a transferable method along the line of “observe → judge → explain.”
3	Change: action from the individual to the world	*Autumn Dusk in the Mountains; Yu Casts the Nine Tripod Cauldrons; The Giant’s Garden*	Integrate causal chains and value judgments from the perspective of how actions trigger change.
4	Balance: an eternal theme of growth	*Qingping Yue: Village Life; Jingwei Fills the Sea; “Wonderful!” and “Terrible!”*	Build a thematic explanation around balancing emotions and evaluations in the process of growing up.

Each thematic cluster was implemented across three 40-minute lessons. The first two lessons in each cluster focused on guided reading, text comprehension, and identification of key details across the three linked texts. The third lesson served as the integrative lesson, in which students compared the three texts, returned to textual evidence, and prepared for cross-text written expression. The complete instructional sequence therefore included 12 lessons across 4 weeks. The intervention and comparison classes received the same number of lessons and the same total instructional time.

In the intervention class, the integrative lesson in each thematic cluster used scaffolded role-play instruction. In this study, scaffolded role-play refers to role-based classroom discussion supported by role cards, probing-question chains, evidence checkpoints, and turn-taking rules. Role cards provided students with speaking positions and textual perspectives. Probing-question chains moved students from initial claims back to textual support. Evidence checkpoints required students to locate specific textual details before confirming or revising a statement. Turn-taking rules were used to regulate classroom pacing. Each integrative lesson followed four stages: situational introduction and task framing, role-based dialogue and negotiated expression, returning to the texts to retrieve evidence and compare similarities and differences, and thematic synthesis with expressive output. A typical integrative lesson lasted 40 min, including 5 min for text review and task framing, 5 min for reading role cards and assigning roles, 15 min for group or whole-class role-based discussion, 8 min for evidence checking and teacher follow-up, and 7 min for written synthesis or oral-to-written preparation. Before offering an interpretation, students were required to introduce evidence using sentence stems such as “The text states…” or “In one of the texts, I noticed…”.

The comparison class followed the school’s regular reading-instruction routine across the same four thematic clusters. Classroom activities included reading aloud for comprehension, question-based discussion, teacher explanation, practice, and summary. The third lesson of each thematic cluster was also used to review the three texts and prepare students for the subsequent cross-text writing task. The amount of time allocated to discussion and written-expression preparation was broadly comparable to that in the intervention class. Classroom interaction in the comparison class was organized mainly through teacher questioning, student responses, and whole-class discussion, without role cards, probing-question chains, mandatory evidence checkpoints, or role-based turn-taking rules. Both classes studied the same thematic clusters and texts, received the same lesson duration and total instructional time, and completed the same posttest writing task.

The scripts used in the intervention class were first drafted by ChatGPT-5.2 and then reviewed by the teacher before classroom use. Script development included three stages: generation, review, and implementation. In the generation stage, a unified prompt template was used across the four thematic units. The template included grade level, text titles, teacher-generated content cues, the unit integrative objective, evidence checkpoints, role number, maximum speaking turns, and required output format. For each thematic unit, the model generated one candidate script at a time, including role cards, situational task framing, probing-question chains, evidence-return prompts, and turn-taking arrangements. The output format required short role speeches, explicit links to textual evidence, and questions guiding students from initial claims to textual support and cross-text comparison. The ChatGPT interface used for lesson preparation did not provide platform-side sampling parameters such as temperature or top-p. Replicability was therefore supported through a fixed prompt template, consistent input fields, repeated output structure, teacher review records, and archived examples of draft and revised scripts.

In the review stage, the teacher checked the AI-generated scripts line by line using a practical review checklist. The checklist focused on textual or source accuracy, language difficulty for Grade 3 students, classroom expression and value orientation, and whether script length fit lesson pacing. The teacher also checked whether the scripts included cross-text comparison prompts, thematic synthesis prompts, and dialogue turns that could be linked back to specific textual evidence. Role speeches that were too long, unclear source references, and overly broad probing questions were compressed or revised. For example, the prompt “Tell us what you think about this character’s wisdom” was revised as “Which sentence or detail in the text shows this character’s wisdom? Explain your judgment based on that detail.” This revision provided a clearer evidence entry point for discussion. In the implementation stage, active roles were limited to 4–6 students, whole-class dialogue to 2–3 rounds, and each round included at least one evidence checkpoint and a sentence stem for returning to the text. To reduce cross-condition contamination, separate lesson plans and classroom materials were used for the two classes. Intervention-specific role cards, probing-question chains, evidence checkpoints, cue cards, and role-based turn-taking rules were used only in the intervention class. The teacher followed the predetermined instructional sequence and recorded completion of the planned stages after each lesson. The inputs, outputs, and checkpoints of ChatGPT-5.2 script generation, teacher review, and classroom implementation are shown in Table 2. The full prompt template, output schema, teacher review checklist, and examples before and after teacher revision are provided in the Supplementary material.

**TABLE 2:** ChatGPT-5.2 script generation, teacher review, and classroom implementation workflow.

Stage	Input / Output	Key checkpoints
Generation	Grade level, three texts, thematic objective, evidence points, role set and max turns, output format → Draft script	Whether role cards define the evidence scope; whether the follow-up question chain drives comparison and synthesis; whether each turn includes a “tie-back” prompt
Review	Draft → Finalized script	Factual alignment with the texts; age-appropriate language; sound value orientation; controllable pacing/rhythm; complete prompts for comparison and synthesis
Implementation	Finalized script → Classroom activity	4–6 roles; 2–3 turns; evidence points required each turn; prescribed tie-back sentence patterns

### 3.4 Measures

After the full instructional sequence, students completed the cross-text integrative writing task followed by the post-intervention learning-experience questionnaire. The focal writing task was administered only at the posttest stage. For the writing task, students selected one of the four thematic units they had studied and wrote approximately 400 Chinese characters to explain how the three texts jointly expressed the theme of that unit. They were also required to compare at least two texts in terms of similarities and differences and to support their claims with specific textual details. The distribution of selected thematic units was recorded by class to check whether there were clear differences between the two classes in topic choice. The selected thematic unit was recorded for each student before scoring and was used to examine topic-selection balance between the two classes. Because some thematic clusters could include small cell counts, Fisher’s exact test was used to compare the distribution of selected thematic units across the intervention and comparison classes. Topic choice was used only as a design-transparency check and was not included as a scoring criterion.

Cross-text integrative writing was assessed using a four-dimension, four-level scoring rubric. The four dimensions were thematic integration, textual evidence, comparison and transfer, and expressive structure. Each dimension was scored from 1 to 4, yielding a total score ranging from 4 to 16. The rubric was informed by research on multi-text comprehension and cross-document integration, especially work on source tracking, content-source integration, and claim-evidence coordination (Perfetti et al., 1999; Rouet and Britt, 2011; Stadtler and Bromme, 2014), as well as source-based writing assessment and the Chinese language curriculum’s emphasis on evidence-supported expression. Thematic integration assessed whether students could construct a shared interpretation across the three texts. Textual evidence assessed whether students used identifiable textual details to support their claims. Comparison and transfer assessed whether students could establish connections among texts and extend the synthesized meaning. Expressive structure assessed the clarity and coherence of written organization. Two raters were trained with sample texts before formal scoring and independently scored all responses while blinded to students’ class membership. Interrater reliability was evaluated using the intraclass correlation coefficient (Shrout and Fleiss, 1979). If the two raters differed by more than one level on any dimension, or by more than two points on the total score, the response entered a review procedure, and the final score was determined through rater discussion based on the rubric.

The post-intervention learning-experience questionnaire was administered after the writing task. The questionnaire used a 5-point Likert scale and included 28 items across six dimensions: engagement and interest, comprehension and evidence awareness, cross-text integration and transfer, strategies and self-efficacy, perceived task difficulty and pressure, and acceptability. The perceived task difficulty and pressure dimension recorded students’ subjective perceptions of task difficulty, uncertainty about where to begin, classroom pace, and pressure during task completion. Dimension scores were calculated as item means, and reverse-coded items were recoded before composite scores were computed. The questionnaire items were developed for the classroom context of the present study, drawing on constructs related to multi-text learning, evidence-based reading discussion, classroom engagement, strategy use, self-efficacy, task burden, and technology-supported instruction. Chinese language education researchers and an experienced elementary Chinese teacher reviewed the items for content relevance, age-appropriate wording, and classroom interpretability. No separate pilot test was conducted before the formal classroom administration; instead, the expert and teacher review was used to check item relevance, wording clarity, age appropriateness, and classroom interpretability before the questionnaire was administered. Internal consistency was evaluated using Cronbach’s α (Cronbach, 1951). Given the sample size and the age of the students, the questionnaire was used mainly to describe students’ perceived classroom experience after the intervention.

Table 3 lists the six dimensions and corresponding items of the post-intervention learning-experience questionnaire. The items covered classroom participation, textual understanding, evidence use, cross-text connection, strategic confidence, task pressure, and instructional acceptability, and were used to supplement the account of students’ subjective experience of the classroom activities.

**TABLE 3:** Online questionnaire items (5-point Likert scale: 1 = strongly disagree, 5 = strongly agree).

Dimension	Item no.	Item statement
C Engagement and interest	C1	During this period, Chinese language classes made me more willing to participate in speaking or discussions.
	C2	I can stay focused more easily in class and am less likely to get distracted.
	C3	I am more interested in the texts and am willing to keep reading or learn more.
	C4	I feel the learning approach in these classes is more interesting than before.
	C5	I am willing to cooperate with classmates to complete tasks during activities.
D Comprehension and evidence awareness	D1	I can clearly explain what these texts are mainly about.
	D2	I can identify the key details in the texts.
	D3	When answering questions or expressing a viewpoint, I go back to the text to find evidence.
	D4	I can distinguish my own guesses or feelings from what is explicitly stated in the text.
	D5	I can clearly articulate the experience and wisdom reflected in the texts.
	D6	I feel my understanding of the texts is deeper than before.
E Cross-text integration and transfer	E1	I can find commonalities among the three texts within the same thematic cluster.
	E2	I can state at least one similarity and one difference and explain the reasons.
	E3	I can connect the three texts and summarize a more comprehensive thematic understanding.
	E4	I can apply methods from the texts to new learning situations.
	E5	I can connect the texts to my own life experience and give specific examples in writing or speaking.
F Strategies and self-efficacy	F1	I feel I can organize my ideas clearly and express them orally or in writing.
	F2	If I don’t understand something, I know what to do.
	F3	I feel more confident about completing comprehension-based short writing tasks or expression assignments.
	F4	I feel I have become more proactive in Chinese language learning.
G Perceived task difficulty and pressure	G1	The Chinese learning tasks during this period were at an appropriate difficulty level for me. (reverse-coded when computing the perceived difficulty/pressure composite)
	G2	During classroom activities, I often don’t know where to start. (negative)
	G3	I feel the activity pace is too fast, and I can’t quite keep up. (negative)
	G4	I feel very nervous or under a lot of pressure when completing tasks. (negative)
H Acceptability	H1	I hope Chinese language classes will continue to use similar learning approaches in the future.
	H2	I think this approach can help more classmates understand the texts better.
	H3	I think the teacher’s guidance is important.
	H4	I feel this approach has made at least one clear improvement in my Chinese language learning.

The questionnaire also included two open-ended questions, asking students which activity component they found most helpful and which aspect they found most difficult or least comfortable. Open-ended responses and representative writing excerpts were used to support the interpretation of the quantitative results. Writing excerpts were selected after scoring, with a focus on differences in evidence use, cross-text comparison, and thematic synthesis. The selection of excerpts considered score level, class membership, and relevance to the scoring dimensions, and all identifiable student information was removed. Chinese excerpts that needed to be translated into English were first translated by the teacher and then checked by the research team.

### 3.5 Data analysis

Data analysis began with data organization and quality checking. The study first summarized participant flow and examined missing data, duplicate submissions, out-of-range responses, clearly implausible questionnaire completion times, and straight-line response patterns. Before scoring, writing responses were checked for task relevance, mainly to identify texts that were clearly too short or deviated from the writing requirements. For the post-intervention learning-experience questionnaire, item means were calculated for each dimension, and reverse-coded items were recoded before being included in the corresponding dimension scores. Internal consistency was evaluated using Cronbach’s α.

Between-class differences in writing performance were analyzed using independent-samples t tests, with equal-variance t tests or Welch’s t tests selected according to Levene’s test results. For the post-intervention learning-experience questionnaire, mean scores were compared across dimensions, and Mann-Whitney U tests were used as robustness checks for Likert-type scores. When multiple comparisons were conducted within the same family of outcomes, the Holm-Bonferroni method was used to adjust the significance level. Mean differences were reported with 95% confidence intervals, and Cohen’s d was reported as the standardized mean-difference effect size. For the main writing outcome, a 95% confidence interval for Cohen’s d was also reported. Supplementary ANCOVA models were reported with partial η^2^.

To control for students’ prior Chinese language achievement, midterm Chinese language scores were entered as a covariate in the supplementary ANCOVA models. These models examined between-class differences in the writing total score and in each rubric dimension. Before interpreting the model results, covariate-outcome linearity, homogeneity of regression slopes, residual distribution, homogeneity of variance, and influential cases were checked, and partial η^2^ was reported as the effect-size estimate. Because the two instructional conditions corresponded to two intact classes, the statistical results mainly represented exploratory between-class differences after student-level adjustment, while allowing for the possible involvement of class-contextual factors in interpretation. The post-intervention learning-experience questionnaire was used as a self-report measure of students’ classroom experience after the instructional sequence. It mainly described students’ perceived classroom participation, textual understanding, evidence awareness, cross-text integration, strategy use, task pressure, and acceptability, and was considered together with the writing posttest results to describe classroom implementation. Given the valid analytic sample size of 54 students, the questionnaire analysis was limited to dimension-level between-class comparisons and descriptive interpretation.

**FIGURE 1:**
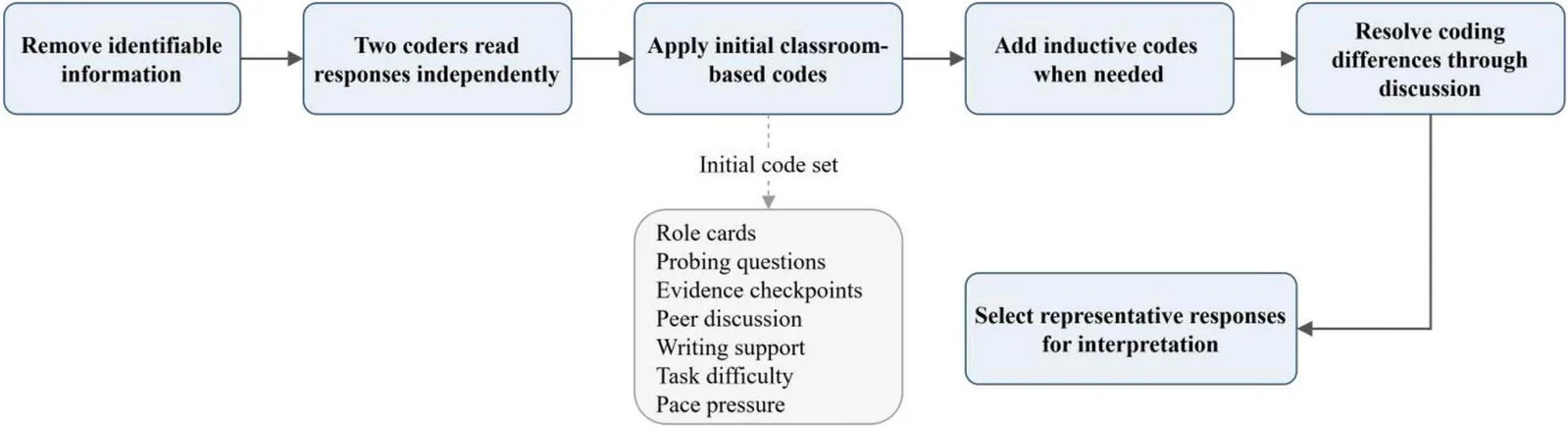
Structured coding workflow for open-ended questionnaire responses.

Open-ended questionnaire responses were organized according to the structured coding workflow shown in Figure 1. After identifiable information was removed, two coders independently read all responses and grouped them using initial categories aligned with the classroom activities. New categories were added when needed, coding disagreements were resolved through discussion, and representative responses were selected to support the interpretation of students’ classroom experiences.

## 4 Results

### 4.1 Data preparation

The initial sample included 56 students, with 28 students in the intervention class and 28 in the comparison class. After data screening, two students were excluded from subsequent analyses because they did not provide complete valid data. The final analytic sample therefore included 54 students, with 27 students in the intervention class and 27 in the comparison class. Among the retained cases, no duplicate submissions, out-of-range questionnaire values, clearly abnormal completion times, or repeated selection of the same response option were identified. Writing responses were checked for task relevance before scoring. No response was excluded for being clearly too short or deviating from the writing task, and writing scores did not cluster at the highest or lowest end of the scale.

Midterm Chinese language scores were used to describe students’ general Chinese language achievement before the instructional period. The intervention class had a mean score of 82.7 (SD = 8.4), and the comparison class had a mean score of 81.9 (SD = 8.1). Welch’s t test showed no statistically significant difference between the two classes, *t*(51.9) = 0.36, *p* = 0.718. This indicator reflected students’ general Chinese language performance; their baseline level on the focal cross-text integrative writing task was not measured separately.

The distribution of selected thematic clusters was also examined before scoring. In the intervention class, the numbers of students selecting Clusters 1–4 were 8, 6, 7, and 6, respectively. The corresponding numbers in the comparison class were 5, 8, 7, and 7. Fisher’s exact test for the 2 × 4 table indicated no clear between-class difference in topic-selection distribution, *p* = 0.843. This result suggested that the two classes did not show an obvious imbalance in the thematic clusters selected for the posttest writing task.

Writing responses were independently scored by two raters. Interrater reliability for the total writing score was ICC(2, k) = 0.87. ICC values for the four rubric dimensions ranged from 0.78 to 0.85, indicating acceptable agreement for thematic integration, textual evidence, comparison and transfer, and expressive structure (Shrout and Fleiss, 1979). Following commonly used interpretive guidelines, ICC values between 0.75 and 0.90 were treated as indicating good interrater reliability. The total-score ICC of 0.87 and the dimension-level ICC range of 0.78–0.85 therefore suggested good agreement between the two raters (Koo and Li, 2016). Internal consistency for the post-intervention learning-experience questionnaire dimensions was as follows: engagement and interest, α = 0.86; comprehension and evidence awareness, α = 0.88; cross-text integration and transfer, α = 0.85; strategies and self-efficacy, α = 0.82; perceived task difficulty and pressure, α = 0.76; and acceptability, α = 0.84. Statistical tests were selected according to the results of distributional checks, variance-homogeneity tests, and outlier inspection, including Welch’s t tests, equal-variance t tests, and Mann–Whitney U robustness checks.

### 4.2 Writing posttest differences

**FIGURE 2:**
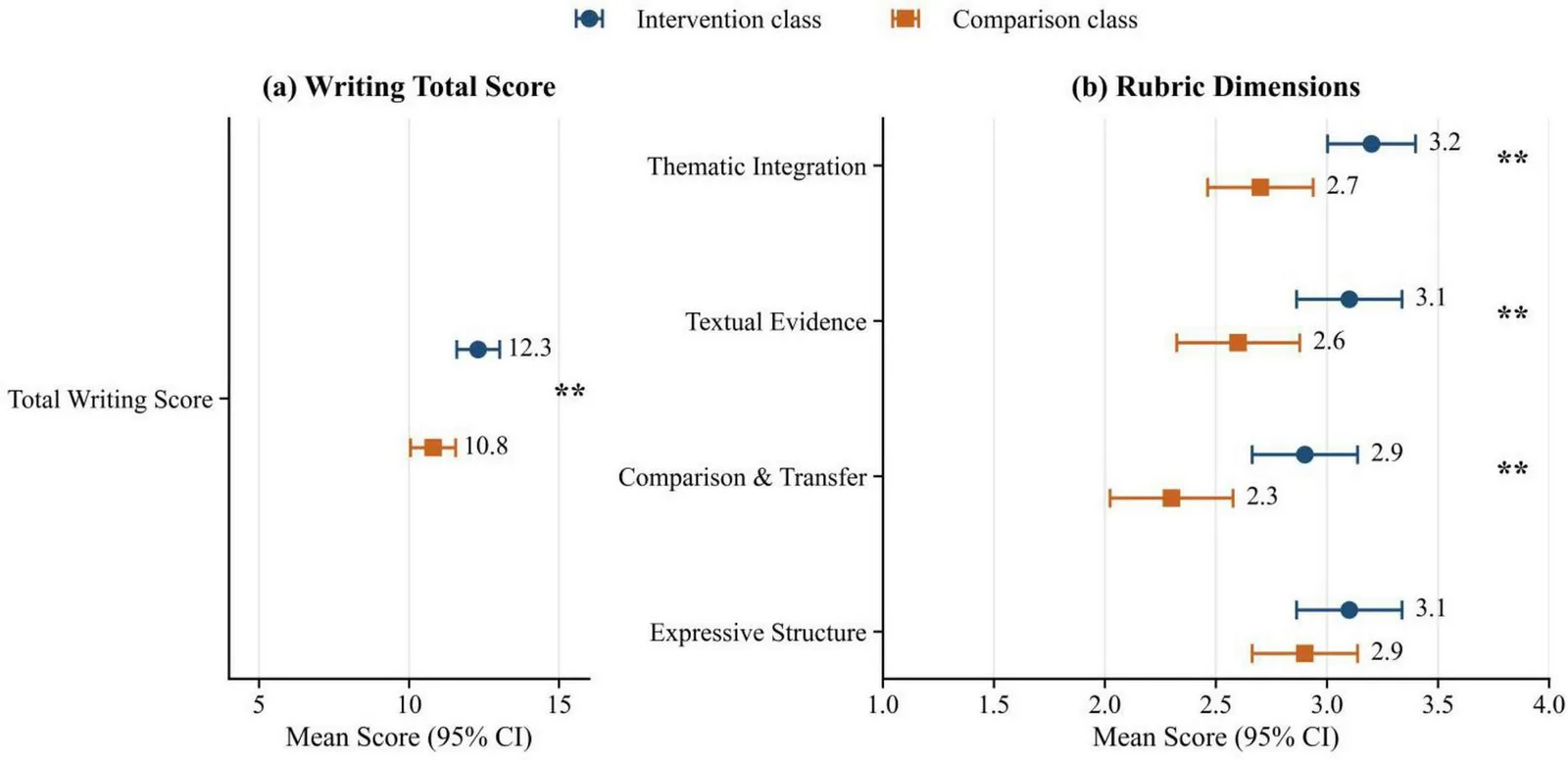
Between-class differences in writing performance. Points show group means and error bars show 95% confidence intervals for the writing total score and four rubric dimensions. **indicates statistically significant group differences retained after Holm-Bonferroni correction for rubric dimensions, *p* < 0.01.

Table 4 and Figure 2 show that the intervention class showed a higher total score on the writing posttest than the comparison class. The intervention class had a mean total writing score of 12.3 (SD = 1.8), whereas the comparison class had a mean score of 10.8 (SD = 1.9). The mean difference between the two classes was 1.5 points, 95% CI for the mean difference = [0.49, 2.51]. Welch’s t test showed that this difference was statistically significant, Welch’s *t*(51.8) = 2.98, *p* = 0.004, Cohen’s d = 0.81, 95% CI for Cohen’s d = [0.25, 1.37]. A supplementary ANCOVA using midterm Chinese language scores as the covariate produced a similar result, *F*(1,51) = 8.72, *p* = 0.005, partial η^2^ = 0.146, with a 95% CI for the adjusted mean difference = [0.47, 2.39]. Model diagnostics indicated an approximately linear relationship between the covariate and the writing outcome. The class × covariate interaction was not significant, *F*(1,50) = 0.42, *p* = 0.520, and residual plots did not show clear heteroscedasticity or influential cases. Because each instructional condition was represented by only one intact class, these student-level tests could not fully separate the instructional condition from unmeasured class-level influences. The writing results were therefore interpreted as exploratory between-class differences after student-level adjustment for prior Chinese language achievement.

**TABLE 4:** Between-class differences in total writing score and rubric dimensions (*N* = 54).

Measure (Range)	Intervention class M (SD)	Comparison class M (SD)	Test	*P*	Cohen’s d	95% CI for mean difference
Total writing score (4–16)	12.3 (1.8)	10.8 (1.9)	Welch’s *t*(51.8) = 2.98	0.004	0.81	[0.49, 2.51]
Thematic integration (1–4)	3.2 (0.5)	2.7 (0.6)	*t*(52) = 3.25	0.002	0.87	[0.19, 0.81]
Textual evidence (1–4)	3.1 (0.6)	2.6 (0.7)	*t*(52) = 2.99	0.004	0.80	[0.16, 0.84]
Comparison and transfer (1–4)	2.9 (0.6)	2.3 (0.7)	*t*(52) = 3.09	0.003	0.83	[0.21, 0.99]
Expressive structure (1–4)	3.1 (0.6)	2.9 (0.6)	*t*(52) = 1.30	0.199	0.35	[−0.11, 0.51]

Further examination of the four rubric dimensions showed that the intervention class had higher posttest scores than the comparison class in thematic integration, textual evidence, and comparison and transfer. For thematic integration, the intervention class had a mean score of 3.2 (SD = 0.5), whereas the comparison class had a mean score of 2.7 (SD = 0.6), *t*(52) = 3.25, *p* = 0.002, Cohen’s *d* = 0.87, 95% CI for the mean difference = [0.19, 0.81]. For textual evidence, the intervention class had a mean score of 3.1 (SD = 0.6), compared with 2.6 (SD = 0.7) in the comparison class, *t*(52) = 2.99, *p* = 0.004, Cohen’s d = 0.80, 95% CI for the mean difference = [0.16, 0.84]. For comparison and transfer, the intervention class had a mean score of 2.9 (SD = 0.6), compared with 2.3 (SD = 0.7) in the comparison class, *t*(52) = 3.09, *p* = 0.003, Cohen’s d = 0.83, 95% CI for the mean difference = [0.21, 0.99]. After Holm-Bonferroni correction, the between-class differences in these three dimensions remained statistically significant, suggesting that the observed posttest differences were concentrated in how students extracted themes, used textual evidence, and established cross-text connections.

The expressive structure dimension showed a different pattern. The intervention class had a mean score of 3.1 (SD = 0.6), whereas the comparison class had a mean score of 2.9 (SD = 0.6), *t*(52) = 1.30, *p* = 0.199, Cohen’s d = 0.35, 95% CI for the mean difference = [−0.11, 0.51]. This dimension did not reach statistical significance, suggesting limited between-class differences in the clarity and coherence of written organization. Taken together, the total-score and dimension-level results indicate that the higher performance of the intervention class was mainly reflected in theme construction, evidence use, and cross-text comparison, all of which are directly related to integrative understanding, whereas the difference in general expressive structure was less pronounced.

### 4.3 Learning-experience questionnaire differences

**FIGURE 3:**
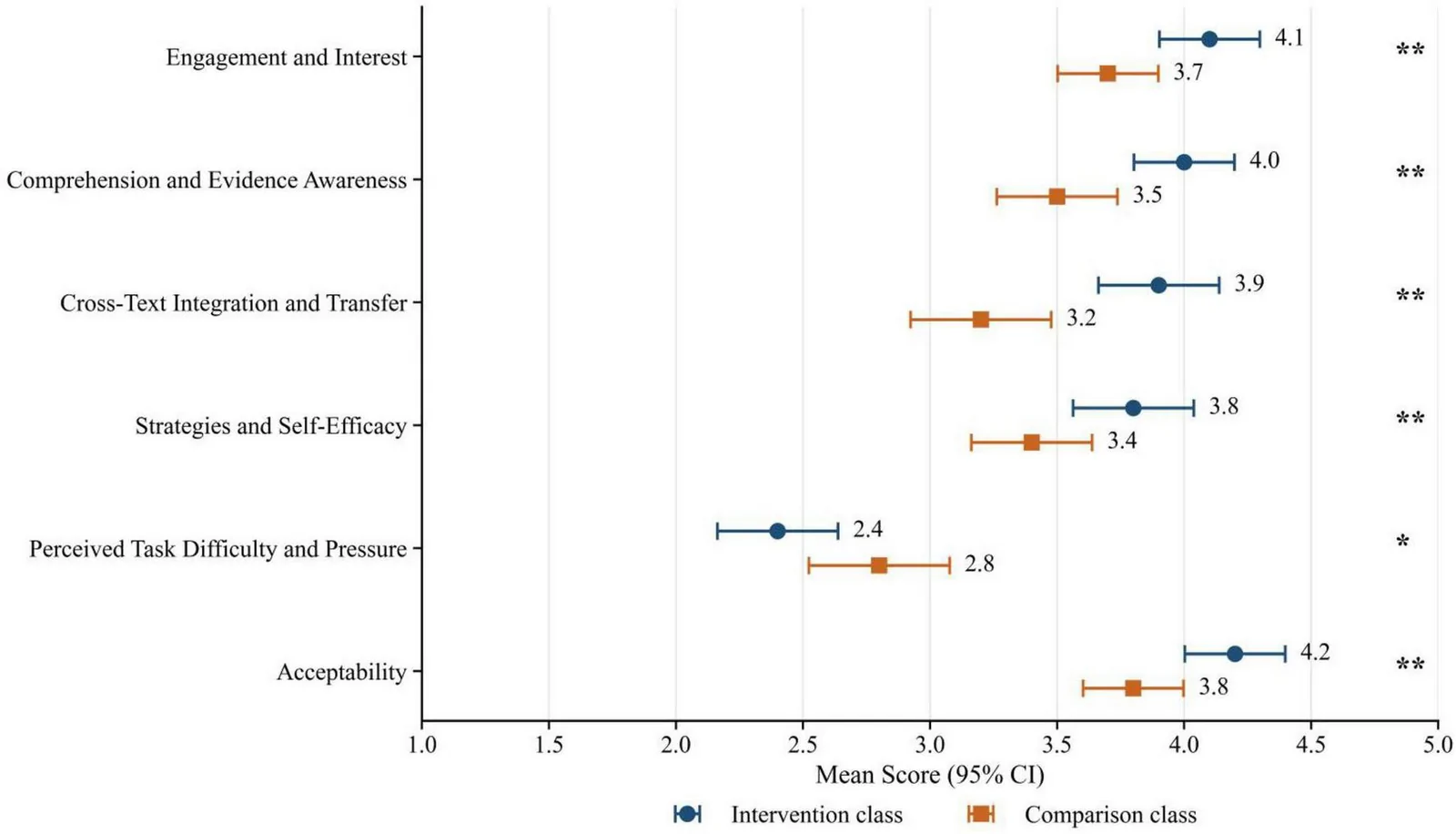
Between-class differences in post-intervention learning-experience questionnaire dimensions. Points indicate group means, and error bars indicate 95% confidence intervals for group means. Higher scores indicate stronger agreement with the corresponding dimension, except for perceived task difficulty and pressure, where higher scores indicate greater subjective burden. Asterisks indicate Holm-Bonferroni-adjusted significance levels across the six questionnaire dimensions: *adjusted *p* < 0.05, **adjusted *p* < 0.01.

Table 5 and Figure 3 report the between-class comparisons for the post-intervention learning-experience questionnaire. The questionnaire was administered after the instructional sequence and was treated as a self-report measure of students’ classroom experience. Its results describe students’ perceived participation, textual understanding, evidence awareness, cross-text integration, strategy use, task pressure, and instructional acceptability, rather than process-level changes observed during classroom learning. The intervention class had higher mean scores than the comparison class on five dimensions: engagement and interest, comprehension and evidence awareness, cross-text integration and transfer, strategies and self-efficacy, and acceptability. The largest difference was observed in cross-text integration and transfer, with a mean score of 3.9 (SD = 0.6) in the intervention class and 3.2 (SD = 0.7) in the comparison class, *t*(52) = 3.89, *p* < 0.001, Cohen’s d = 1.04, 95% CI for the mean difference = [0.34, 1.06]. Comprehension and evidence awareness also showed a large difference, with a mean score of 4.0 (SD = 0.5) in the intervention class and 3.5 (SD = 0.6) in the comparison class, *t*(52) = 3.56, *p* < 0.001, Cohen’s d = 0.95, 95% CI for the mean difference = [0.22, 0.78]. At the level of students’ self-reports, these results show that students in the intervention class more often reported connecting multiple texts, returning to the texts to locate evidence, and forming a more complete thematic understanding.

**TABLE 5:** Between-class differences in post-intervention learning-experience questionnaire dimensions (*N* = 54).

Dimension	Intervention class M (SD)	Comparison class M (SD)	Test	*P*	Cohen’s d	95% CI for mean difference
Engagement and interest	4.1 (0.5)	3.7 (0.5)	*t*(52) = 3.25	0.002	0.87	[0.15, 0.65]
Comprehension and evidence awareness	4.0 (0.5)	3.5 (0.6)	*t*(52) = 3.56	< 0.001	0.95	[0.22, 0.78]
Cross-text integration and transfer	3.9 (0.6)	3.2 (0.7)	*t*(52) = 3.89	< 0.001	1.04	[0.34, 1.06]
Strategies and self-efficacy	3.8 (0.6)	3.4 (0.6)	*t*(52) = 2.85	0.006	0.76	[0.12, 0.68]
Perceived task difficulty and pressure	2.4 (0.6)	2.8 (0.7)	*t*(52) = −2.22	0.031	−0.59	[−0.76, −0.04]
Acceptability	4.2 (0.5)	3.8 (0.5)	*t*(52) = 3.25	0.002	0.87	[0.15, 0.65]

Engagement and interest, strategies and self-efficacy, and acceptability also showed higher self-reported levels in the intervention class. For engagement and interest, the intervention class had a mean score of 4.1 (SD = 0.5), compared with 3.7 (SD = 0.5) in the comparison class, *t*(52) = 3.25, *p* = 0.002, Cohen’s d = 0.87, 95% CI for the mean difference = [0.15, 0.65]. For strategies and self-efficacy, the intervention class had a mean score of 3.8 (SD = 0.6), compared with 3.4 (SD = 0.6) in the comparison class, *t*(52) = 2.85, *p* = 0.006, Cohen’s d = 0.76, 95% CI for the mean difference = [0.12, 0.68]. For acceptability, the intervention class had a mean score of 4.2 (SD = 0.5), compared with 3.8 (SD = 0.5) in the comparison class, *t*(52) = 3.25, *p* = 0.002, Cohen’s d = 0.87, 95% CI for the mean difference = [0.15, 0.65]. The perceived task difficulty and pressure dimension showed the opposite direction: the intervention class had a mean score of 2.4 (SD = 0.6), whereas the comparison class had a mean score of 2.8 (SD = 0.7), *t*(52) = −2.22, *p* = 0.031, Cohen’s d = −0.59, 95% CI for the mean difference = [−0.76, −0.04]. This self-reported pattern suggests that the intervention class perceived lower task difficulty and pressure while also reporting higher participation, evidence awareness, and instructional acceptability.

The Mann–Whitney U robustness checks showed the same directional pattern as the parametric tests. After Holm–Bonferroni correction, the between-class differences across the six questionnaire dimensions remained statistically significant. Overall, students in the intervention class reported more positive classroom participation, textual understanding, evidence awareness, cross-text connection, strategic confidence, and instructional acceptability, while reporting lower task difficulty and pressure. These findings are interpreted as perceived differences in classroom learning experience after the instructional sequence. Students’ age, the novelty of the role-play activities, classroom atmosphere, teacher expectancy, social desirability bias, general response tendencies, and parental assistance during questionnaire completion may all have shaped the questionnaire responses. The study did not include an external criterion measure, repeated questionnaire administration, or an independent observation record that could be used to estimate or adjust for these response influences. For this reason, no formal sensitivity analysis or statistical bias correction was applied to the questionnaire data. Accordingly, the questionnaire results are used to supplement the account of students’ subjective classroom experience and are not treated as evidence of stable changes in learning strategies, self-efficacy, or cross-text integration ability.

### 4.4 Representative writing excerpts

Table 6 presents two student writing excerpts to show how the scoring differences appeared in specific texts. The excerpts were selected from student writings after scoring, and unusually high- or low-scoring texts were avoided. The selection focused on evidence use, cross-text comparison, and thematic synthesis, with the aim of supplementing the writing features indicated by the quantitative results reported above. To make the link between the writing excerpts and the scoring results explicit, the excerpts were interpreted in relation to the four rubric dimensions. Thematic integration referred to whether the student formed a shared thematic judgment across the three texts. Textual evidence referred to whether the student used specific textual details to support claims. Comparison and transfer referred to whether the student explained similarities, differences, or transferable meanings across texts. Expressive structure referred to whether the response showed clear paragraph organization, sentence-level connection, and development of ideas. This cross-reference was used to connect the representative excerpts with the dimension-level results, where the clearest between-class differences appeared in thematic integration, textual evidence, and comparison and transfer, while expressive structure showed a weaker difference.

**TABLE 6:** Illustrative student writing excerpts (selected).

Sample (group)	Writing excerpt (selected)	Evidence tie-back performance	Thematic integration and transfer performance
Sample A (intervention class)	“From *A Night Mooring by Maple Bridge, Borrowing Arrows with Straw Boats, and Venice’s Little Boat*, I see three different kinds of wisdom. Zhang Ji, on a fishing boat by Maple Bridge, hears the bell from Hanshan Temple and slowly settles the loneliness of travel—this is the wisdom of making peace with oneself; Zhuge Liang borrows arrows with straw boats by accurately reading the weather and understanding people’s minds—this is the wisdom of strategic planning; the boatmen in Venice handle their small boats so skillfully that they become indispensable to the city—this is the wisdom of applying one’s skills to everyday life. […] Last time I was lost while building LEGO, and later I learned to analyze the situation: I sorted the pieces first, then worked step by step, and finally completed the model.”	Under a shared theme, the excerpt repeatedly ties back to key details from all three texts and uses those details directly to support judgments; the evidence-claim linkage is clear.	Connects the three texts into a single thematic line and provides a concrete real-life transfer example; comparison is mainly achieved through parallel presentation of different types of wisdom.
Sample B (comparison class)	“The wisdom on the waterways is a kind of wisdom of innovation, unity, and dedication. *Borrowing Arrows with Straw Boats* tells me the importance of innovative thinking; I also see the power of unity and cooperation; *Venice’s Little Boat* tells me the importance of dedication. […] In daily life, I also help the community promote waste sorting, pick up litter, and pass on environmental awareness to people around me.”	Mentions the text titles but extracts fewer key scenes/details; claims are mostly general summaries, with comparatively weak evidence support.	Reads more like listing moral takeaways from each text in sequence; commonalities/differences across texts are not developed; the transfer example is concrete, but its linkage to textual evidence is not tight enough.

The excerpt from the intervention class organizes the three texts around “different kinds of wisdom.” The student first places *A Night Mooring by Maple Bridge, Borrowing Arrows with Straw Boats*, and *Venice’s Little Boat* within a shared thematic frame, and then cites textual details such as Zhang Ji hearing the bell from Hanshan Temple, Zhuge Liang completing the strategy of borrowing arrows by judging the weather and people’s minds, and the Venetian boatmen skillfully steering their boats. These details do not remain at the level of plot retelling. Instead, they are used to distinguish “the wisdom of reconciling with oneself,” “the wisdom of planning,” and “the wisdom of applying skills to everyday life.” In terms of the rubric dimensions, the excerpt shows thematic integration by placing the three texts within the same interpretive framework, textual evidence by using identifiable details from each text, and comparison and transfer by presenting different forms of wisdom and extending them to a new life situation. Its expressive structure is sufficient for the reader to follow the student’s reasoning, while the comparison is still mainly realized through parallel examples. In the later transfer to daily life, the student writes that when building a LEGO model, they first sorted the pieces and then completed the model step by step, which continues the earlier line of understanding about analyzing a situation and solving a problem.

The excerpt from the comparison class also attempts to summarize the value of the texts and extend the theme to daily life. It uses general terms such as “innovation,” “unity,” and “dedication,” and mentions text titles such as *Borrowing Arrows with Straw Boats* and *Venice’s Little Boat*. However, it provides fewer specific scenes or character actions to support these judgments. The three texts appear more like several parallel lessons in the writing, and their similarities, differences, and shared theme are not fully developed. The later example about waste sorting is relatively concrete, but its connection with the preceding textual analysis is weak. Mapped to the rubric dimensions, the excerpt shows an attempt at thematic integration but relies mainly on broad value labels. Textual evidence is limited because claims are not closely tied to specific textual details. Comparison and transfer are also less developed because the text-to-text relationships and the connection between the texts and the life example remain loose. Expressive structure is not the main weakness, since the response is generally readable; the more evident limitation lies in the development of evidence, textual relationships, and the basis for transfer.

## 5 Discussion

Viewed from the writing-rating dimensions, the intervention-class advantage was mainly concentrated in aspects closely related to cross-text integration. Students in the intervention class performed better in thematic integration, textual evidence, and comparison and transfer, while the difference in expressive structure was limited. This pattern fits the nature of multi-text integration tasks, in which students need to identify a shared theme across texts, select relevant details, compare textual information, and support synthesized judgments with evidence. Recent research on multiple-text comprehension has also emphasized that integration is shaped by task context, source use, affect, culture, and technology (Nash et al., 2025). In the intervention class, role-based discussion, evidence checkpoints, and probing questions directed students back to the texts before they moved into written expression. This attention to sources and evidence is consistent with work showing that task instructions can shape how learners move among source information, evidence paragraphs, and written integration in multiple-text reading (Guan and Lin, 2025). The writing excerpts showed a similar pattern: the intervention-class excerpt organized the three texts under different forms of “wisdom” and connected each judgment with textual details, while the comparison-class excerpt relied more on broad value labels. Since AI-assisted preparation, role-play, evidence prompts, and structured discussion were implemented together, the results should be understood as the effect of an integrated classroom arrangement, not the effect of any single component.

The weaker difference in expressive structure suggests that the intervention provided stronger support for what students wrote about than for how they organized the final written text. Role cards, probing questions, and evidence checkpoints helped students generate claims, locate evidence, and connect texts during discussion. However, written organization also requires paragraph development, sentence-to-sentence cohesion, balance between general claims and specific details, and revision of language. This result may reflect a transfer gap between oral discussion support and written discourse construction. Role-play can help students produce ideas and evidence in interaction, but sentence-level cohesion and paragraph-level organization usually require additional composing, modeling, and revision opportunities. This is consistent with research suggesting that prompts are most useful when they are closely aligned with learner actions and task demands (Thomann and Deutscher, 2025), as well as studies showing that students need explicit support to use feedback when moving from response generation to revision (Hornstein et al., 2025). Future instruction could therefore add short model paragraphs, sentence-connection exercises, and revision checkpoints after role-based discussion, so that students have clearer support for turning discussion outcomes into coherent written texts.

The learning-experience questionnaire helps explain how students perceived the classroom process. Students in the intervention class reported higher engagement, evidence awareness, perceived cross-text integration and transfer, strategies and self-efficacy, and acceptability, while reporting lower task difficulty and pressure. Role positions may have given students a clearer entry point into discussion, question chains may have reduced uncertainty about how to continue speaking, and evidence checkpoints made the requirement to return to the text more visible. The lower perceived difficulty suggests that, from students’ own perspective, the added demands of comparing texts and citing evidence did not necessarily increase task burden. These results should be read as self-reported classroom experience after the instructional sequence. They do not by themselves show stable changes in learning strategies, self-efficacy, or cross-text integration ability. Such changes would need to be examined with pretest–posttest data, delayed measures, and classroom-process records.

From the perspective of teacher preparation, generative AI was used mainly to produce initial drafts of instructional materials. ChatGPT helped generate role cards, situational tasks, and probing questions, but the drafts still required teacher selection and revision before classroom use. Some outputs were too broad, too long, weakly connected to the source texts, or too difficult for Grade 3 students. Some value-related expressions also needed adjustment to fit the language and developmental level of an elementary Chinese classroom. The teacher therefore had to check the scripts against the texts, simplify language, shorten dialogue sequences, clarify evidence links, and add evidence checkpoints. In this sense, AI reduced part of the initial drafting work, but it did not replace the teacher’s judgment about textual accuracy, student comprehension, classroom rhythm, or age-appropriate expression. For lower-grade classrooms, source checking and language adaptation remain central. The study also limited AI input to instructional design information, such as text titles, teaching objectives, role requirements, script-format requirements, and evidence checkpoints. Student data, identifiable writing samples, full textbook passages, scanned pages, and long verbatim textbook excerpts were not entered into ChatGPT.

Overall, the study suggests that teacher-vetted AI-assisted preparation can be integrated into ordinary Chinese language teaching when it is tied to clear evidence-based classroom routines. The role-play activities were useful not because they made the class more active in a general sense, but because they gave students a structured way to speak from a textual position, check evidence, compare texts, and prepare for writing. The findings therefore point to a practical direction for linked-text reading instruction: students may benefit when oral discussion, evidence use, and written synthesis are arranged as a continuous process. At the same time, the classroom arrangement in this study still gave more support to theme construction and evidence use than to extended written organization. Future work should examine how this kind of instructional design can be combined with paragraph modeling, revision feedback, and delayed writing tasks, so that students are supported not only in forming cross-text judgments but also in developing more coherent written explanations.

## 6 Limitations

This study was conducted in two intact Grade 3 classes at a public primary school in Tianjin, so the findings should be understood in relation to the authentic classroom context. Although the two classes were taught by the same teacher and used the same instructional materials, schedule, learning objectives, and posttest task, factors such as class background, reading experience, and discussion habits may still have influenced the results. The study did not include a pretest of cross-text integrative writing, and midterm Chinese language scores were used mainly as a general reference for students’ Chinese language achievement. With a valid analytic sample of 54 students, the analyses focused mainly on between-class comparisons and supplementary ANCOVA. The learning-experience questionnaire was developed for the present classroom context and was not separately pilot-tested, so its results mainly reflect students’ self-reported classroom experiences. Students’ age, classroom atmosphere, and parental technical assistance may have shaped their responses. The instructional activities lasted four thematic units and 12 lessons, which helped reduce the influence of a single-session novelty effect, although this issue still needs to be further examined through delayed measures and classroom observations. AI was used mainly to generate initial drafts of instructional materials, while the teacher was responsible for screening, revising, and adapting the materials for classroom use. Student data and full copyrighted textbook content were not entered into ChatGPT. Future studies could further examine the instructional approach and its applicability across more schools, grades, teachers, and text types.

## 7 Conclusion

This study examined an integrated instructional package in the context of Grade 3 Chinese linked reading. The package combined teacher-reviewed AI-generated materials, scaffolded role-play, evidence checkpoints, probing-question chains, structured turn-taking, and cross-text writing. The intact-class posttest comparison showed that the intervention class scored higher than the comparison class on the total writing score. The differences were mainly reflected in thematic integration, textual evidence, and comparison and transfer, whereas the difference in expressive structure was relatively limited. This result suggests that the integrated instructional package was associated with stronger performance in returning to the texts to locate evidence, comparing cross-text information, and forming synthesized judgments. Support for paragraph development, sentence-to-sentence cohesion, and overall discourse organization still needs to be further strengthened.

During implementation, ChatGPT was used mainly at the teacher-preparation stage to generate initial drafts of role cards, situational tasks, and probing questions. Classroom use depended on the teacher’s review and revision of textual accuracy, age appropriateness, value orientation, and instructional pacing. The findings suggest that, when AI-assisted draft materials are selected and revised by the teacher and embedded within explicit teacher-led scaffolds, the integrated instructional package may provide a feasible classroom approach to organizing evidence-oriented cross-text discussion and writing. Because this study was based on a posttest-only comparison of two intact classes, did not collect a prior cross-text integrative writing baseline, and used questionnaire data derived from students’ self-reports, the findings should be understood as preliminary evidence from an authentic classroom setting. Future studies could expand the class sample, include a pretest and delayed measurement of cross-text integrative writing, and incorporate classroom discussion records, evidence-citation turns, and writing-revision processes to further examine the classroom conditions under which teacher-vetted AI-assisted preparation, scaffolded discussion, and cross-text writing may support students’ integrative expression across new text combinations.

## Data Availability

The original contributions presented in this study are included in this article/Supplementary material, further inquiries can be directed to the corresponding author.
