# Supplementary material for: Scaffolded role-play for cross-text integrative writing in elementary Chinese: a teacher-vetted AI-drafted materials classroom study
Source: Front Psychol. 2026 Aug 7;17:1837762. doi: 10.3389/fpsyg.2026.1837762 (PMC13493221; doi:10.3389/fpsyg.2026.1837762)
Supplement: Supplementary file 1 [file Supplementary_file_1.DOCX]

Supplementary Material

# 1 **Supporting Information**

## 1.1 Supporting Information S1. LLM Script Package (JSON)

### 1.1.1 S1.1 JSON Overall Structure (Suggested filename: scripts_bundle.json)

{

"package_meta": {

"package_id": "LLM_RolePlay_Scripts_PrimaryChinese_v1",

"grade_band": "Primary (upper elementary)",

"language": "zh-CN",

"purpose": "Cross-text thematic integration (three-text set reading) with evidence-anchored role-play",

"core_constraints": {

"role_count": "4-6",

"rounds": "2-3",

"evidence_rule": "Evidence first, then claim",

"no_student_data": true

}

},

"scripts": []

}

### 1.1.2 S1.2 Script Field Descriptions

- group_id: Theme group ID
- texts: Three texts (title/genre/evidence cues only, no original text excerpts)
- theme_goal: Thematic integration objective (aligned with rubric "Thematic Integration")
- evidence_anchors: Evidence cue checklist (aligned with rubric "Text Evidence")
- roles: Role cards (stance, responsibilities, available evidence scope, prohibited behaviors)
- dialogue_plan: Round-based dialogue script (each round must include "evidence callback" and "comparison/integration")
- question_ladder: Follow-up question chain (from clarification → evidence → comparison → integration → transfer)
- hard_rules: Classroom constraints (rounds, speaking length, evidence sentence patterns, etc.)
- teacher_review_log_template: Review record template (factual accuracy, age-appropriateness, values, pacing, evidence constraints)

### 1.1.3 S1.3 Four Theme Group Script Examples (Following Same JSON Structure)

Script G1：舟楫上的智与行（枫桥夜泊 / 草船借箭 / 威尼斯的小艇）

{
 "group_id": "G1",
 "theme_title": "Wisdom in action: decisions, skills, and situational judgment",
 "texts": [
 {
 "text_id": "T1",
 "title": "枫桥夜泊",
 "genre": "poetry",
 "evidence_cues": [
 "夜景与声音线索（钟声/江面/客船等）",
 "情绪从漂泊到安放的转折",
 "以景写情的表达方式"
 ]
 },
 {
 "text_id": "T2",
 "title": "草船借箭",
 "genre": "narrative",
 "evidence_cues": [
 "对时机与天气的判断",
 "对对方心理与行动的预判",
 "计划执行中的关键动作与结果"
 ]
 },
 {
 "text_id": "T3",
 "title": "威尼斯的小艇",
 "genre": "expository/narrative description",
 "evidence_cues": [
 "小艇与城市生活的关系",
 "船夫技艺与操作细节",
 "功能与价值的说明路径"
 ]
 }
 ],
 "theme_goal": {
 "driving_question": "What kinds of wisdom do the three texts show, and how are they similar or different?",
 "expected_synthesis": "Integrate three texts into one theme line: situational judgment + skilled action + emotional self-regulation, supported by text-specific details."
 },
 "evidence_anchors": {
 "evidence_sentence_starters": [
 "在（文本名）里写到……这说明……",
 "我注意到（细节线索），所以我认为……",
 "从（情节/画面/做法）可以看出……"
 ],
 "compare_starters": [
 "相同点是……不同点在于……",
 "A更像……而B更像……因为……",
 "如果把三篇放在同一主题下……"
 ],
 "transfer_starters": [
 "在我的生活/学习中，我也遇到……我可以用……",
 "这个主题给我的启发是……我准备这样做……"
 ]
 },
 "roles": [
 {
 "role_id": "R1",
 "name": "Evidence Keeper",
 "stance": "Insists on text-based support",
 "tasks": [
 "Ask classmates to name the text and the concrete cue",
 "Stop over-general moral statements",
 "Record which evidence supports which claim"
 ],
 "must_do": ["Every turn asks: which text? which cue?"],
 "must_avoid": ["Inventing details not implied by cues"]
 },
 {
 "role_id": "R2",
 "name": "Comparer",
 "stance": "Organizes similarities/differences",
 "tasks": [
 "Frame a comparison axis (emotion/strategy/skill)",
 "Produce at least one similarity and one difference per round"
 ],
 "must_do": ["Use at least one compare_starter per round"],
 "must_avoid": ["Listing texts separately without linking"]
 },
 {
 "role_id": "R3",
 "name": "Theme Builder",
 "stance": "Builds a single theme line",
 "tasks": [
 "Propose a theme statement that can cover all three texts",
 "Refine theme based on evidence and comparison"
 ],
 "must_do": ["End each round with a one-sentence theme update"],
 "must_avoid": ["Theme too vague (e.g., 'be good')"]
 },
 {
 "role_id": "R4",
 "name": "Transfer Connector",
 "stance": "Connects to life/learning with specificity",
 "tasks": [
 "Give one concrete transfer example",
 "Explain how the example maps to a specific text cue"
 ],
 "must_do": ["Transfer must cite at least one cue"],
 "must_avoid": ["Transfer unrelated to the theme line"]
 }
 ],
 "hard_rules": {
 "roles_in_class": 4,
 "rounds": 2,
 "turn_length_limit_seconds": 25,
 "evidence_required_each_turn": true,
 "teacher_intervention_triggers": [
 "No evidence cue mentioned",
 "Only moral slogans",
 "Off-topic personal stories"
 ]
 },
 "dialogue_plan": [
 {
 "round": 1,
 "goal": "Generate claims with anchored cues",
 "steps": [
 {
 "speaker": "Theme Builder",
 "prompt": "Give a tentative theme line that might cover the three texts. Keep it specific.",
 "expected_move": "Theme hypothesis"
 },
 {
 "speaker": "Evidence Keeper",
 "prompt": "Name one cue from one text that supports the theme. Say the text and the cue.",
 "expected_move": "Evidence anchoring"
 },
 {
 "speaker": "Comparer",
 "prompt": "State one similarity and one difference across two texts using cues.",
 "expected_move": "Comparison"
 },
 {
 "speaker": "Transfer Connector",
 "prompt": "Give one concrete life example and map it back to one cue.",
 "expected_move": "Transfer with mapping"
 }
 ],
 "round_output": "Theme line v1 + 3 evidence-claim pairs + 1 comparison axis"
 },
 {
 "round": 2,
 "goal": "Synthesize and prepare writing plan",
 "steps": [
 {
 "speaker": "Comparer",
 "prompt": "Choose a comparison axis (emotion/strategy/skill). Assign each text a distinct contribution.",
 "expected_move": "Structured cross-text map"
 },
 {
 "speaker": "Evidence Keeper",
 "prompt": "For each text, point to one cue that matches the assigned contribution.",
 "expected_move": "Evidence list for writing"
 },
 {
 "speaker": "Theme Builder",
 "prompt": "Write a final theme sentence that includes the comparison axis.",
 "expected_move": "Final synthesis"
 },
 {
 "speaker": "Transfer Connector",
 "prompt": "Draft one transfer sentence that clearly follows from the final theme and cites a cue.",
 "expected_move": "Transfer sentence for essay ending"
 }
 ],
 "round_output": "Final theme line + writing outline (3 paragraphs evidence map + transfer ending)"
 }
 ],
 "question_ladder": {
 "clarify": ["你说的智慧具体指什么？用一句话界定。"],
 "evidence": ["你依据的是哪一篇？哪个画面/动作/要点？"],
 "compare": ["两篇的相同点是什么？不同点因为什么？"],
 "synthesize": ["把三篇放在一起，这条主线能不能解释每一篇？"],
 "transfer": ["你生活中的例子如何对应到某个证据线索？"]
 },
 "teacher_review_log_template": {
 "factual_alignment": "Check cues match text content; remove invented details",
 "age_appropriateness": "Simplify wording; avoid abstract jargon",
 "value_safety": "Avoid inappropriate moralizing or sensitive content",
 "tempo_control": "Compress to 2 rounds; cap roles at 4-6",
 "evidence_binding": "Ensure each turn has cue + claim mapping"
 }
}

Script G2：观察：认知世界的起点（暮江吟 / 王戎不取道旁李 / 松鼠）

{
 "group_id": "G2",
 "theme_title": "Observation to inference: seeing, judging, explaining",
 "texts": [
 { "text_id": "T1", "title": "暮江吟", "genre": "poetry", "evidence_cues": ["颜色与光影变化", "静态画面与感受", "用景物带出理解"] },
 { "text_id": "T2", "title": "王戎不取道旁李", "genre": "narrative", "evidence_cues": ["看到现象（果实状态）", "推断原因（为何不摘）", "验证结果（与他人对照）"] },
 { "text_id": "T3", "title": "松鼠", "genre": "expository", "evidence_cues": ["外形/习性特征", "说明顺序与分类", "例子支撑说明点"] }
 ],
 "theme_goal": {
 "driving_question": "How do the three texts show observation leading to understanding?",
 "expected_synthesis": "A shared route: notice → select cues → infer/explain → express clearly."
 },
 "roles": [
 { "role_id": "R1", "name": "Spotter", "tasks": ["Name concrete observable cues"], "must_do": ["Always start with a cue"], "must_avoid": ["Abstract claims without cues"] },
 { "role_id": "R2", "name": "Reasoner", "tasks": ["Explain how cue supports inference"], "must_do": ["Use because linking"], "must_avoid": ["Jumping to conclusion"] },
 { "role_id": "R3", "name": "Organizer", "tasks": ["Build an explanation order"], "must_do": ["Propose structure for writing"], "must_avoid": ["Scattered points"] },
 { "role_id": "R4", "name": "Checker", "tasks": ["Ask for source text and verify consistency"], "must_do": ["Which text? which cue?"], "must_avoid": ["Accepting vague answers"] }
 ],
 "hard_rules": { "roles_in_class": 4, "rounds": 2, "evidence_required_each_turn": true },
 "dialogue_plan": [
 {
 "round": 1,
 "goal": "Cue-to-inference practice across texts",
 "steps": [
 { "speaker": "Spotter", "prompt": "Pick one cue from one text and say it plainly.", "expected_move": "Cue" },
 { "speaker": "Reasoner", "prompt": "Explain what we can infer from that cue and why.", "expected_move": "Inference with because" },
 { "speaker": "Checker", "prompt": "Confirm the cue belongs to which text and is not invented.", "expected_move": "Source check" },
 { "speaker": "Organizer", "prompt": "Turn the cue+inference into one sentence for writing.", "expected_move": "Sentence draft" }
 ]
 },
 {
 "round": 2,
 "goal": "Cross-text synthesis + writing outline",
 "steps": [
 { "speaker": "Organizer", "prompt": "Build a 3-part outline: observation → inference → explanation.", "expected_move": "Outline" },
 { "speaker": "Checker", "prompt": "For each part, demand one cue from a different text.", "expected_move": "3 cues mapping" },
 { "speaker": "Reasoner", "prompt": "State a similarity and a difference in how texts use observation.", "expected_move": "Compare" },
 { "speaker": "Spotter", "prompt": "Give one real-life example where observation helped you decide.", "expected_move": "Transfer" }
 ]
 }
 ]
}

Script G3：改变：从个体到世界的行动（山居秋暝 / 禹铸九鼎 / 巨人的花园）

{
 "group_id": "G3",
 "theme_title": "Action and change: causes, consequences, and values",
 "texts": [
 { "text_id": "T1", "title": "山居秋暝", "genre": "poetry", "evidence_cues": ["景物变化与氛围", "人物活动的点染", "以景见情/价值取向"] },
 { "text_id": "T2", "title": "禹铸九鼎", "genre": "legend/historical narrative", "evidence_cues": ["行动目标与象征意义", "过程与凝聚", "结果如何影响共同体"] },
 { "text_id": "T3", "title": "巨人的花园", "genre": "fairy tale", "evidence_cues": ["巨人行为变化", "环境与他人反应变化", "结局指向的价值判断"] }
 ],
 "theme_goal": {
 "driving_question": "What changes happen because of actions, and what values do they imply?",
 "expected_synthesis": "One line: action reshapes environment/relationships; texts differ in tone and evidence type."
 },
 "roles": [
 { "role_id": "R1", "name": "Cause Mapper", "tasks": ["Link action → consequence"], "must_do": ["Use 'because/therefore'"], "must_avoid": ["Only retell plot"] },
 { "role_id": "R2", "name": "Value Judge", "tasks": ["Explain value implied by change"], "must_do": ["Tie value to a cue"], "must_avoid": ["Moral slogans"] },
 { "role_id": "R3", "name": "Cross-Text Linker", "tasks": ["Find cross-text parallel"], "must_do": ["One similarity+one difference"], "must_avoid": ["Three separate summaries"] },
 { "role_id": "R4", "name": "Evidence Keeper", "tasks": ["Demand cue"], "must_do": ["Which text? which cue?"], "must_avoid": ["Accepting vague claims"] }
 ],
 "hard_rules": { "roles_in_class": 4, "rounds": 2, "evidence_required_each_turn": true },
 "dialogue_plan": [
 {
 "round": 1,
 "goal": "Build action→change chains in each text",
 "steps": [
 { "speaker": "Cause Mapper", "prompt": "Choose one text and state an action and its consequence.", "expected_move": "Chain" },
 { "speaker": "Evidence Keeper", "prompt": "Name the cue that shows the action or consequence.", "expected_move": "Evidence" },
 { "speaker": "Value Judge", "prompt": "What value does this change suggest? Keep it tied to the cue.", "expected_move": "Value with evidence" },
 { "speaker": "Cross-Text Linker", "prompt": "Match a similar chain in another text and one key difference.", "expected_move": "Compare" }
 ]
 },
 {
 "round": 2,
 "goal": "Synthesize a theme + prepare writing structure",
 "steps": [
 { "speaker": "Cross-Text Linker", "prompt": "Propose a synthesis sentence covering all three texts.", "expected_move": "Theme line" },
 { "speaker": "Evidence Keeper", "prompt": "List one cue per text that supports the synthesis.", "expected_move": "3-cue support" },
 { "speaker": "Cause Mapper", "prompt": "Draft a paragraph order: Text1→Text2→Text3 with cause-change links.", "expected_move": "Outline" },
 { "speaker": "Value Judge", "prompt": "Add a transfer ending: how will you act differently in life?", "expected_move": "Transfer" }
 ]
 }
 ]
}

Script G4：平衡：成长的永恒课题（清平乐·村居 / 精卫填海 / 精彩极了和糟糕透了）

{
 "group_id": "G4",
 "theme_title": "Balance in growth: emotions, persistence, and evaluation",
 "texts": [
 { "text_id": "T1", "title": "清平乐·村居", "genre": "poetry", "evidence_cues": ["生活画面与情绪基调", "细节呈现的温度", "整体氛围的表达方式"] },
 { "text_id": "T2", "title": "精卫填海", "genre": "myth", "evidence_cues": ["目标与坚持", "重复行动的象征", "情感力量的表达"] },
 { "text_id": "T3", "title": "精彩极了和糟糕透了", "genre": "narrative", "evidence_cues": ["两种评价的对照", "人物心理变化", "理解与平衡的形成"] }
 ],
 "theme_goal": {
 "driving_question": "How do the three texts help us understand balance in growth?",
 "expected_synthesis": "Balance is not 'neutral'; it is coordinating emotion, persistence, and feedback."
 },
 "roles": [
 { "role_id": "R1", "name": "Emotion Reader", "tasks": ["Name emotional cues"], "must_do": ["Cue→emotion→meaning"], "must_avoid": ["Empty praise"] },
 { "role_id": "R2", "name": "Persistence Analyst", "tasks": ["Explain persistence with evidence"], "must_do": ["Why persistence matters here"], "must_avoid": ["Blind perseverance slogans"] },
 { "role_id": "R3", "name": "Feedback Balancer", "tasks": ["Compare positive/negative evaluation"], "must_do": ["One similarity+one difference"], "must_avoid": ["Take one-sided stance"] },
 { "role_id": "R4", "name": "Evidence Keeper", "tasks": ["Enforce source cue"], "must_do": ["Which text? which cue?"], "must_avoid": ["Inventing quotes"] }
 ],
 "hard_rules": { "roles_in_class": 4, "rounds": 2, "evidence_required_each_turn": true },
 "dialogue_plan": [
 {
 "round": 1,
 "goal": "Build evidence-based statements for each text",
 "steps": [
 { "speaker": "Emotion Reader", "prompt": "Pick a cue from the poem and explain the emotion it conveys.", "expected_move": "Emotion with cue" },
 { "speaker": "Persistence Analyst", "prompt": "Pick a cue from the myth and explain what persistence looks like.", "expected_move": "Persistence with cue" },
 { "speaker": "Feedback Balancer", "prompt": "Pick a cue from the narrative about evaluation and explain the balance.", "expected_move": "Feedback balance with cue" },
 { "speaker": "Evidence Keeper", "prompt": "Check each statement: which cue, which text, what claim?", "expected_move": "Cue-claim mapping" }
 ]
 },
 {
 "round": 2,
 "goal": "Integrate into one theme line + transfer",
 "steps": [
 { "speaker": "Feedback Balancer", "prompt": "Combine the three into one theme sentence about balance in growth.", "expected_move": "Synthesis" },
 { "speaker": "Evidence Keeper", "prompt": "Provide one cue per text that supports the synthesis.", "expected_move": "Evidence list" },
 { "speaker": "Emotion Reader", "prompt": "State a similarity and a difference across texts in tone/evidence type.", "expected_move": "Compare" },
 { "speaker": "Persistence Analyst", "prompt": "Give one concrete transfer example tied to one cue.", "expected_move": "Transfer" }
 ]
 }
 ]
}

**Script G1: Wisdom and Action on the Water (Mooring by Night at Maple Bridge / Borrowing Arrows with Thatched Boats / The Little Boats of Venice)**

Due to length constraints, complete JSON examples are provided in the original markdown file. Each script follows this structure:

- group_id: Unique identifier (e.g., "G1")
- theme_title: Overarching theme connecting the three texts
- texts: Array of three text objects with evidence cues
- theme_goal: Driving question and expected synthesis
- evidence_anchors: Sentence starters for evidence, comparison, and transfer
- roles: 4-6 role definitions with specific tasks and constraints
- hard_rules: Classroom implementation constraints
- dialogue_plan: 2-3 rounds of structured discussion

## 1.2 Supporting Information S2. Prompt Inventory (Table)

### 1.2.1 Table S1. Prompt Template Summary (For Reproducing Script Generation and Review)

| Prompt ID | Purpose | Input Variables (Examples) | Key Constraints | Output Format |
| --- | --- | --- | --- | --- |
| P1 | Generate classroom role-play script draft | Grade, three text titles and genres, theme objective, evidence cue list, number of roles, maximum rounds | No original textbook quotes; each round must include evidence callback; include comparison and integration; age-appropriate language | JSON (with roles / dialogue_plan / hard_rules) |
| P2 | Factual and age-appropriate review suggestions | P1 script JSON + teacher review standards | Remove fabricated details; compress rounds; replace difficult words; neutral value statements | JSON differential suggestions (patch) |
| P3 | Evidence binding check | Script JSON | Whether each step includes cue+claim; whether text_id is tagged | Output issue list + revision suggestions |
| P4 | Pacing and workload control | Script JSON + class time (minutes) | Roles 4–6; rounds 2–3; each person each round ≤25 seconds | Output compressed dialogue_plan |
| P5 | Post-class writing scaffold generation | Final theme line + 3 evidence cues + comparison axis | Generate writing outline (not write for students) | JSON (outline + sentence_starters) |

## 1.3 Supporting Information S3. Full Prompt Templates

*The following prompts can be copied and used directly. It is recommended to use P1 for generation, then P2–P4 for review and compression, and finally P5 to generate writing scaffolds. To reproduce the experimental process, it is recommended to save the input and output of each run as a JSON log.*

**P1 Script Generation Prompt (Template)**

You are designing a primary-school Chinese reading lesson using role-play to support cross-text thematic integration.

**TASK:**

Create a classroom script package for a three-text set reading lesson.

**INPUTS:**

- Grade: {GRADE}

- Text set (3 items): {TEXTS_WITH_GENRE}

- Theme goal (one sentence): {THEME_GOAL}

- Evidence cues per text (bullets, no quotes): {EVIDENCE_CUES}

- Constraints: roles={ROLE_COUNT} (4–6), rounds={ROUNDS} (2–3), max_turn_seconds={TURN_SECONDS} (<=25)

- Hard rule: Every speaking turn must include (text name + one concrete cue) before making a claim.

**SAFETY / QUALITY RULES:**

- Do NOT quote the textbook or fabricate lines. Use only cue-level references.

- Age-appropriate wording; avoid abstract jargon.

- Avoid moral slogans. Claims must be linked to cues.

- Include at least one explicit comparison step and one synthesis step.

- Include one transfer step with a concrete example linked back to one cue.

**OUTPUT FORMAT:**

Return JSON only with the following keys: group_id, theme_title, texts, theme_goal, evidence_anchors, roles, hard_rules, dialogue_plan, question_ladder, teacher_review_log_template.

**P2 Teacher Review Prompt (Template)**

You are a teacher reviewer. You will receive a JSON script package. Check it with four lenses: factual alignment, age appropriateness, value safety, tempo control. Return: (1) A short checklist of issues found (bullets). (2) A JSON patch-style suggestion that edits the original JSON minimally.

**P3 Evidence Binding Check Prompt (Template)**

Given a JSON script package, audit evidence binding. Rules: Each step must contain a prompt that forces the student to name (text) + (one cue). Output: missing_binding, fix_suggestions, binding_score (0–100).

**P4 Tempo Compression Prompt (Template)**

Compress the dialogue_plan to fit {TOTAL_MINUTES} minutes. Constraints: roles <= 6, rounds <= 3, each role speaks <= 1 time per round. Keep evidence binding, one comparison, one synthesis, one transfer. Output JSON only with updated hard_rules and dialogue_plan.

**P5 Writing Scaffold Prompt (Template)**

You are generating a writing scaffold (not the essay). Inputs: final_theme_line, comparison_axis, evidence_map, transfer_example_seed. Output JSON with: outline (3 paragraphs + conclusion transfer), sentence_starters (evidence / comparison / synthesis / transfer), a 4-item self-check list aligned to rubric.
